# Managing overlap of primary study results across systematic reviews: practical considerations for authors of overviews of reviews

**DOI:** 10.1186/s12874-021-01269-y

**Published:** 2021-07-07

**Authors:** Carole Lunny, Dawid Pieper, Pierre Thabet, Salmaan Kanji

**Affiliations:** 1 Cochrane Hypertension Group and the Therapeutics Initiative, Department of Anesthesiology, Pharmacology and Therapeutics, University of British Columbia, Vancouver, Canada; 2 Institute for Research in Operative Medicine, Witten/Herdecke University, Cologne, Germany; 3 Hôpital Montfort, Ottawa, Canada; 4 The Ottawa Hospital and Ottawa Hospital Research Institute, Ottawa, Canada

**Keywords:** Overviews of systematic reviews, Meta-review, Overview methodology, Review methods, Reporting, Umbrella review, Evidence synthesis, Overlap, Precision

## Abstract

**Background:**

Overviews often identify and synthesise a large number of systematic reviews on the same topic, which is likely to lead to overlap (i.e. duplication) in primary studies across the reviews. Using a primary study result multiple times in the same analysis overstates its sample size and number of events, falsely leading to greater precision in the analysis. This paper aims to: (a) describe types of overlapping data that arise from the same primary studies reported across multiple reviews, (b) describe methods to identify and explain overlap of primary study data, and (c) present six case studies illustrating different approaches to manage overlap.

**Methods:**

We first updated the search in PubMed for methods from the MOoR framework relating to overlap of primary studies. One author screened the studies titles and abstracts, and any full-text articles retrieved, extracted methods data relating to overlap of primary studies and mapped it to the overlap methods from the MOoR framework. We also describe six case studies as examples of overviews that use specific overlap methods across the steps in the conduct of an overview. For each case study, we discuss potential methodological implications in terms of limitations, efficiency, usability, and resource use.

**Results:**

Nine methods studies were found and mapped to the methods identified by the MOoR framework to address overlap. Overlap methods were mapped across four steps in the conduct of an overview – the eligibility criteria step, the data extraction step, the assessment of risk of bias step, and the synthesis step. Our overview case studies used multiple methods to reduce overlap at different steps in the conduct of an overview.

**Conclusions:**

Our study underlines that there is currently no standard methodological approach to deal with overlap in primary studies across reviews. The level of complexity when dealing with overlap can vary depending on the yield, trends and patterns of the included literature and the scope of the overview question. Choosing a method might be dependent on the number of included reviews and their primary studies. Gaps in evaluation of methods to address overlap were found and further investigation in this area is needed.

**Supplementary Information:**

The online version contains supplementary material available at 10.1186/s12874-021-01269-y.

## Background

Navigating the expanding body of research literature is an increasing challenge for health practitioners, researchers and decision-makers. Global research output as a whole is growing rapidly, and it is estimated that every 9 years, publications in Web of Science double [1]. The number of published systematic reviews being produced yearly is also expanding [2, 3], and duplication of reviews on similar topics is common. For example, Doundoulakis et al. [4] found 57 meta-analyses on direct oral anticoagulants for stroke prevention in atrial fibrillation. Their inclusion criteria were meta-analyses with comprehensive search strategies and risk of bias assessments. If their eligibility criteria had been less restrictive, over 100 meta-analyses would have been found on this topic. Faced with a large volume of systematic reviews on the same topic, healthcare providers need a method to make sense of potentially conflicting, discrepant and overlapping information of varying quality [3].

Overviews of systematic reviews (i.e. umbrella reviews, meta-reviews, reviews of reviews, or reviews of meta-analyses [henceforth called overviews] [5]), offer a solution to this challenge by proposing a method to synthesize the results and conclusions at the systematic review level [6, 7]. Overviews are increasing in volume in response to the growing number of systematic reviews. From 2000 to 2020, 1218 overviews were published, the majority of which (886/1218 [73%]) were published in the most recent 5 year period (2016-2020) [8].

Overviews often identify and synthesise a large number of systematic reviews on the same topic, which is likely to lead to overlap (i.e. duplication) in primary studies across the reviews. For example, a broad overview of 16 natural therapies, found largely to be ineffective, led to changing Australia’s Private Health Insurance Act of 2007 [9]. Overlap can arise when systematic reviews on the same topic include one or more identical primary studies (e.g. randomised control trials (RCTs), cohort and cross-sectional studies). The overlapping data from the same primary studies reported across multiple systematic reviews may include: overlapping risk of bias assessments, overlapping pooled effect estimates across similar outcomes, overlapping meta-analysis results (e.g. I^2^ heterogeneity statistics), or overlapping certainty of the evidence assessments (e.g. Grading of Recommendations, Assessment, Development and Evaluations (GRADE)) [6, 7].

### Example of overlapping primary study data 

**Fig. 1:**
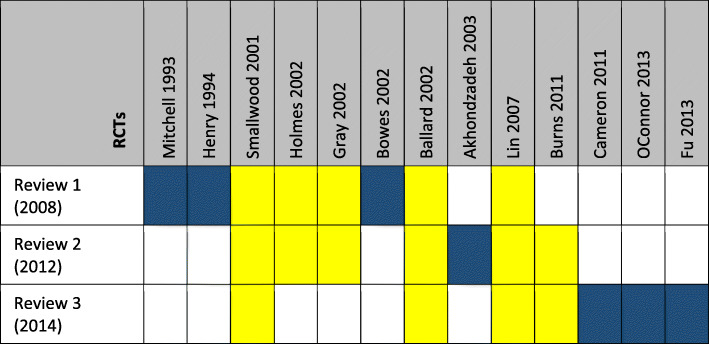
Three reviews included in a fictional overview with overlapping randomised control trials (RCTs)

Synthesising systematic reviews with overlapping primary study data is a challenge for overview authors. As an example, we present three reviews included in a fictional overview, each with a set of RCTs, which are indicated by the coloured boxes in Fig. 1. One of the potential options for dealing with the overlap in RCTs is to base the results on only one systematic review using methodological criteria to select that review, for example, choose the review with the greatest number of trials (i.e. Review 1 with 8 trials). However, this 2008 review is out of date, and leaves out the 4 more recent trials. Alternatively, overview authors could choose Review 2 with the highest quality. However, this high quality review omits 6 trials. A third option is to include the most recent review, Review 3, but again 6 trials would be omitted.

Alternatively, all the reviews could be included, which then involves quantifying the overlap and considering its influence when summarizing the results across the reviews (narratively or statistically). The methods used to deal with overlap of trials as highlighted here, could influence the interpretation of results and conclusions of the overview.

Overlap is a problem of precision related to sampling (i.e. it is not a bias). The inclusion of the same primary study in more than one systematic review gives undue weight to this study. Using a study multiple times in the same analysis overstates its sample size and number of events, falsely leading to greater precision in the analysis. This may impact both narrative description of the results, or a statistical synthesis (e.g. including the results from a primary study more than once in the same meta-analysis).

### Methods development

Mapping, evaluation and development of methods used in overviews has grown over the last 12 years [10–16]. A systematic map of all methods used in overviews, called the MOoR framework, identified nine methods to manage the overlap used across four steps in the conduct of an overview [7, 16]. Since publication of the MOoR framework, methods for overlap have been published [17–19]. These methods are currently being used in practice, and overview guidance has been recently updated [19, 20], but there has been limited evaluation of these methods.

This paper aims to: (a) describe the different types of overlapping data that arise from the same primary studies reported across multiple reviews, (b) describe methods to identify and explain overlap of primary study data, and (c) present six case studies illustrating different approaches to manage overlap.

## Methods

We first updated the search for methods from the MOoR framework [7, 16, 21] relating to overlap of primary studies. We conducted a search in PubMed using the following algorithm: (method*[TI] OR meta-epidemiol*) in combination with the Boolean operator AND, and the search filter for overviews developed by Lunny et al. [5] (Additional file 1: Appendix A). We also conducted forward citation searching on a key study from 2014 [13] dealing with overlap using Google Scholar. Search dates were from January 2016 to March 2020.

We considered articles eligible for inclusion if they described methods used to manage overlapping data across primary studies in overviews of health interventions.

Inclusion criteria:

- Articles describing methods for overviews of systematic reviews of health interventions
- Articles examining methods used in a cross-section or cohort of overviews
- Guidance (e.g. handbooks and guidelines) for undertaking overviews
- Commentaries or editorials that discuss methods for overviews

Exclusion criteria:

- Articles published in languages other than English
- Articles describing methods for network meta-analysis
- Protocols or registered reports
- Articles exclusively about methods for overviews of other review types (i.e. not of interventions)

One author screened the studies titles and abstracts, and any full-text articles retrieved, against the inclusion criteria. One author extracted methods data relating to overlap of primary studies and mapped it to the overlap methods from the MOoR framework (Additional file 1: Appendix B). Characteristics of studies were extracted by one reviewer, as well as the characteristics of the case studies. Results are presented descriptively and in tables.

We also describe six case studies [22–27] as examples of overviews that use specific methods across the steps in the conduct of an overview. The case studies were selectively chosen based on the variety of different approaches used to manage overlap. For each case study, we will discuss potential implications in terms of methodological limitations, efficiency, usability, and resource use.

## Results

### Screening results

**Fig. 2:**
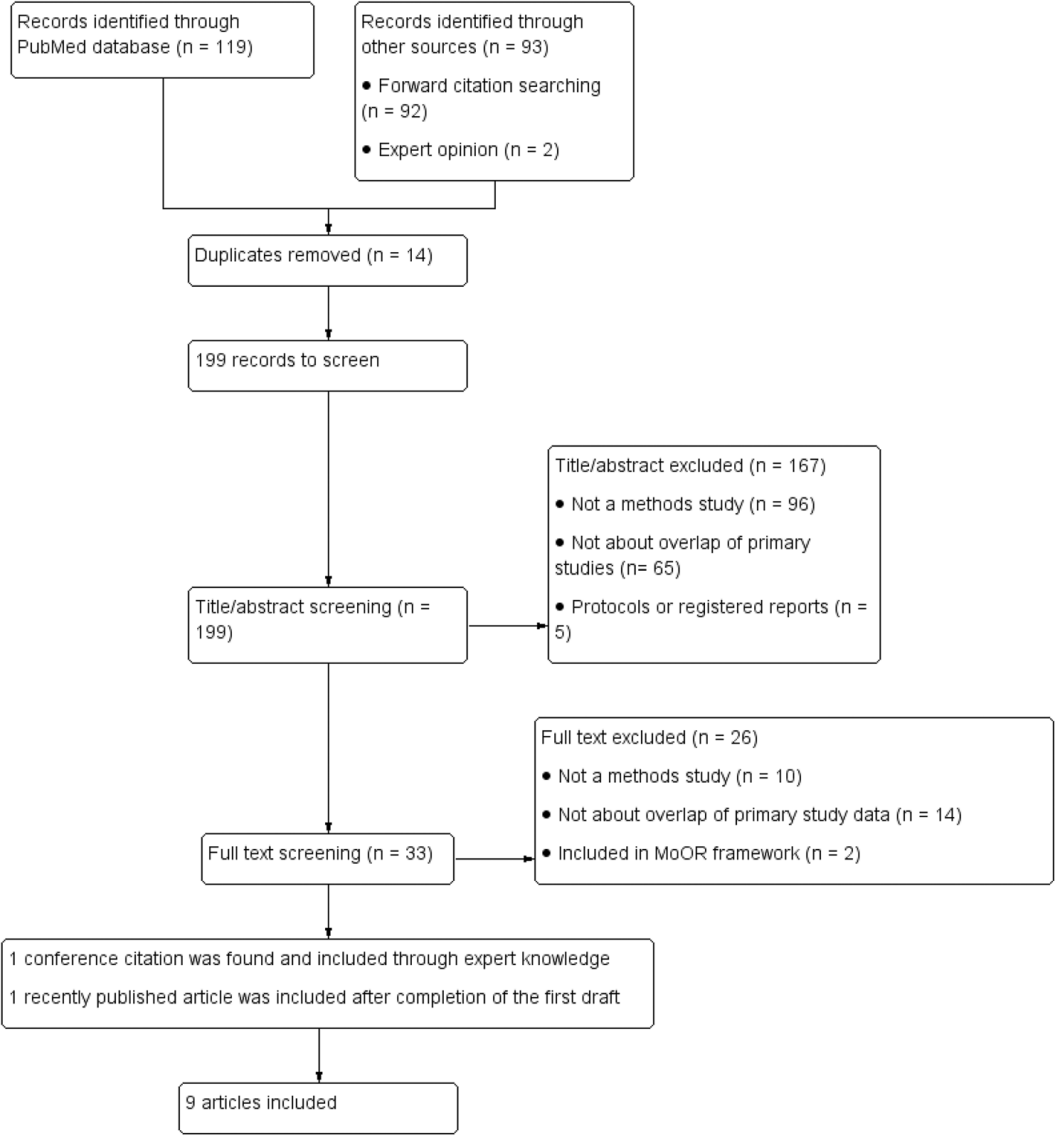
Flowchart of included studies

Our search strategy retrieved 119 unique records, and the forward citation searching retrieved 92 citations (Fig. 2). After deduplication 199 remained, and after screening abstracts and full text, seven were included [17–19, 28–31]. One additional conference citation was found through expert knowledge on the topic [32], and one paper recently published was included after completion of the first draft [33].

### Characteristics of methods studies

Six studies were articles describing methods for overviews, two were guidance documents, and one was an empirical study (Table 1).

**Table 1:** Characteristics of methods studies on overlapping primary study data across reviews

Author Year	Title	Type of study	Method	Objective
**Descriptive studies**				
Ballard 2017 [29]	Risk of bias in overviews of reviews: a scoping review of methodological guidance and four-item checklist	Article describing methods for overviews of systematic reviews of interventions	Scoping review of guidance and methods	Synthesise guidance on overview practice
Bougioukas 2020 [33]	Methods for depicting overlap in overviews of systematic reviews: An introduction to static tabular and graphical displays	Article describing methods for overviews of systematic reviews of interventions	Selective review of papers presenting graphs	Present graphs for visually presenting overlap
Hennessy 2019 [30]	Best practice guidelines and essential methodological steps to conduct rigorous and systematic meta-reviews	Article describing methods for overviews of systematic reviews of interventions	Literature review of methods	Described six steps to address challenges in overviews
Hennessey 2020 [17]	Examining overlap of included studies in meta-reviews: guidance for using the corrected covered area index	Article describing methods for overviews of systematic reviews of interventions	Elaboration of an established method	Described five steps when examining overlap, illustrated through an example
Pollock A. 2017 [18]	Selecting and implementing overview methods: implications from five exemplar overviews	Article examining methods used in a cross-section or cohort of overviews	Elaboration of an established method	Describes methodological challenges of five overviews
Pollock M. 2019 [28]	Chapter V: Overviews of reviews. In Cochrane Handbook for Systematic Reviews of Interventions v 6.0	Guidance for undertaking overviews	Guidance document	Guidance on how and when to assess overlap across primary studies
Pollock M. 2019 [31]	A decision tool to help researchers make decisions about including systematic reviews in overviews of reviews of healthcare interventions	Guidance for undertaking overviews	Decision tool	Systematically conducted seven overviews five times each, making five different decisions about which systematic reviews to include
Pérez-Bracchiglione 2019 [32]	Graphical representation of overlap degree of primary studies in systematic reviews in overviews [abstract OS29.1]	Articles describing methods for overviews of systematic reviews of interventions	Elaboration of an established method	Outlines an overlap assessment tool based on the corrected covered area (CCA [13])
**Empirical study**				
Pollock M. 2019 [19]	The impact of different inclusion decisions on the comprehensiveness and complexity of overviews of reviews of healthcare interventions	Empirical study	Empirical study of an established method	Assessed the impact of five inclusion decisions on the outcome data lost and changed

### Methods studies identified and mapped to the MOoR framework

Nine studies were mapped to the methods identified by the MOoR framework to address overlap (Table 2). Several methods map across four steps in the conduct of an overview – the eligibility criteria step, the data extraction step, the assessment of risk of bias step, and the synthesis step. Seven of the nine studies looked at methods at the eligibility criteria step. One of these studies empirically evaluated the impact of five inclusion decisions on the conduct of an overview [19]. The authors found that when overviews contain overlapping primary studies, selecting a Cochrane systematic review, as opposed to the most recent or highest quality non-Cochrane review, maximized the amount of outcome data included in the overview [19].

**Table 2:** Methods identified from the MOoR Framework mapped to newly identified studies

Step in the conduct of an overview	Methods identified from the MOoR framework [7, 16]	Methods studies	Case studies
Eligibility criteria step	Include all reviews (manage overlap at other stages)	Pollock [28, 31]	Murphy [23] Patnode [27]
	Select one (or more) reviews using pre-specified eligibility criteria	Ballard [29]; Hennessy [17, 30]; Pollock [18]; Pollock [19, 28, 31]	Bidonde [22] Patnode [27] Prousali [24] Thabet [26]
	Select one review from multiple addressing the same question using pre-specified decision rules (e.g. combine one or more eligibility criteria in an algorithm)	Hennessy [17, 30]; Pollock [18]; Pollock [19, 28]	Ryan [25]
	Exclude reviews that do not contain any unique primary studies, when there are multiple reviews	Hennessey [30]; Pollock [18]; Pollock [28]	Ryan [25] (a cut-off of 50% unique primary studies was used)
Data extraction step	Extract all reviews (manage overlap at other stages)	Pollock [19]	Bidonde [22] Patnode [27] Prousali [24] Thabet [26]
	Extract data from only one (or more) reviews using pre-specified eligibility criteria	Pollock [19, 31]	Murphy [23] Patnode [27]
Assessment of risk of bias step	Select one (or more) high quality reviews, or exclude low quality reviews, using pre-specified criteria	Hennessy [17]; Pollock [19, 28]	Murphy [23] Patnode [27] Prousali [24] Ryan [25]
Synthesis and presentation and summary of findings step	Quantifying the amount of overlap (e.g. CCA [13])	Ballard [29]; Hennessy [17, 30]; Pollock [18]; Pollock [19, 28, 31]	Bidonde [22] Murphy [23] Patnode [27] Prousali [24] Ryan [25]^a^ Thabet [26]
	Visually present overlap (e.g. matrix, figures)	Hennessy [17, 30]; Pollock [19, 28, 31]; Bougioukas [33]	Bidonde [22] Murphy [23] Patnode [27] Prousali [24] Thabet
	Select one review (e.g. high quality and comprehensive review using decision rules)	Hennessey [30]	Patnode [27] Ryan [25]
	Use a statistical method (e.g. conduct sensitivity analyses, inflate the variance of the pooled meta-analysis estimate)	Hennessey [30]	Patnode [27]

^a^Cochrane reviews with approximately 50% or more of their studies already captured by Cochrane reviews were excluded

At the eligibility criteria step, one common strategy is to limit the number of included reviews in the overview. This method can be addressed by selecting one, or a subset of systematic reviews from multiple addressing the same question using pre-specified quality criteria (e.g. select systematic reviews that are of high quality) or take a particular methodological approach (e.g. select systematic reviews with meta-analysis of four or more primary studies) (Table 2).

To determine if overlap is present, methods proposed in the data extraction step can allow abstraction of data required for assessment of the extent of the overlap across systematic reviews. Overlapping data from primary studies can then be managed using multiple methods at the synthesis step, including the options to: (a) use decision rules to select results for analysis from one, or a subset of systematic reviews, (b) determine methods for quantifying overlap, or (c) use statistical approaches to deal with overlap. Many methods such as quantifying overlap using the corrected covered area (CCA [13]), or visually examining and presenting overlap of the primary studies across systematic reviews may not directly address the issue but may provide data on the nature and extent of the problem.

Overlap in information can also arise from duplicate risk of bias/quality assessments, or duplicate GRADE outcome assessments. Risk of bias data from the same primary study can differ between what is reported in systematic reviews due to:

- error in data extraction [34]
- data extracted from different sources for the same primary study (e.g. different reports, unpublished data) [35]
- data retrieved/not retrieved by contacting primary study authors [36]
- different tools used to assess risk of bias which leads to missing or inconsistent information about potential biases [37], and/or
- conflicting information reported to support judgements for the same risk of bias tool [38].

Discrepant and overlapping risk of bias assessments across systematic reviews can be resolved by: (a) extracting the risk of bias assessment of primary studies from the included systematic reviews, using data extraction approaches to manage missing, flawed assessments, or discrepant assessments of the same primary study; or (b) re-assessing all primary studies using a common risk of bias tool. The data extraction approaches outlined in the MOoR framework to manage discrepant data across systematic reviews involve retrieving either published or registry reports of the primary studies, or contacting systematic review or primary study authors, or both, for clarification regarding discrepancies [7, 16].

Authors can take additional steps to manage overlapping information and data at the synthesis stage [7, 16]. Two non-statistical methods for resolving overlap in primary studies were identified in the MOoR framework:

- Select the result of one (or a subset of) systematic reviews with or without meta-analysis using a decision rule or a published algorithm [39–41]
- Identify systematic reviews with or without meta-analysis with 25% or more of their research in common and eliminate the one with the fewer studies [11]

Three statistical methods for addressing the overlap in primary study data across systematic reviews were identified in the MOoR framework:

- Conduct sensitivity analyses (e.g. second-order meta-analysis (MA) including all MAs irrespective of overlap compared with second-order MA including only MAs where there is no overlap in primary studies) [11]
- Inflate the variance of the MA estimate [42]; that is, an inflation factor of *J* can be multiplied with the second order MA variance to correct for the underestimated variance estimator.

### Presentation of case studies

Choosing between overlap methods often depends on the type of review being conducted and the clinical topic being investigated. To illustrate how authors used these methods, we now present six case studies to illustrate examples of methods used at different steps in the conduct of an overview, with a commentary on potential implications of the methods in terms of methodological limitations, efficiency, usability, and resource use.

#### 1) Bidonde et al. exercise for adults with fibromyalgia

The overview by Bidonde [22] evaluates physical activity interventions for adults with fibromyalgia with a focus on four outcomes: pain, multidimensional function, physical function and adverse effects. To restrict the number of included reviews, the authors first selected only reviews meeting three or more of Cochrane’s criteria of a systematic review. These were: (a) a focused question (i.e., contains PICO [population, intervention, comparison, outcome] statement); (b) a comprehensive and explicit search (i.e., more than one database and other sources searched, keywords or Mesh terms given); (c) the use of explicit criteria to include and exclude RCTs; (d) explicit methods of extracting and synthesising study findings (quantitative); and (e) inferences made were evidence based.

The authors chose to deal with overlap at the synthesis, presentation and summary of findings step using quantification of the amount of overlap and presenting the results (Table 2). The authors counted 29 (48%) RCTs overlapping across 9 reviews, 31 (52%) of which were ‘unique’ RCTs, and presented the data in tables. For each review, the number of included RCTs was reported, followed by the number of overlapping RCTs between the review and any other reviews. Here is an exemplary quote illustrating this: “[The overview by] Kelley 2010 included seven RCTs: three overlapped with [the overview by] Bidonde, six with [the overview by] Hauser, three with [the overview by] Lima, one with McVeigh, and two with Ramel”.

The methods described here to deal with overlapping primary studies are resource-friendly. The method to restrict inclusion to systematic reviews help reduce the amount of overlap and during the synthesis stage, overlap is quantified. Neither of these methods resolve the problem of overlap, in the same way that judging studies at low or high quality does not resolve the issue of the inclusion of low quality evidence in a review. As with quality appraisal, overlap should be minimised, quantified and used to contextualise the results and conclusions of the overview. Ordering results by amount of overlap may increase the prominence of studies with low overlap, focusing attention on the results that should most influence conclusions. Synthesis of the results of reviews can be limited to those with little or no overlap as a sensitivity analysis.

#### 2) Patnode et al. tobacco cessation in adults

We present the case of an overview by Patnode and colleagues [27], which aimed to compare and synthesise systematic review-level evidence of the effectiveness and safety of pharmacotherapy and behavioral tobacco cessation interventions among adults, including pregnant women and those with mental health conditions. Patnode [27] used nine methods to deal with the overlap in primary studies across 54 included systematic reviews across four steps in the conduct of an overview (Table 2). To manage the overlap in primary studies, the authors chose to exclude non-systematic reviews. At the data extraction and the assessment of risk of bias steps, they chose to exclude all low quality systematic reviews. Quality of the reviews was assessed using the AMSTAR tool [43]. These methods restrict the number of systematic reviews that the authors must analyse at the synthesis step.

At the synthesis step, the authors developed a decision rule. If multiple good quality systematic reviews were identified, the decision rule was applied to determine which systematic review represented the most comprehensive, up-to-date literature base and highest quality to serve as the basis for the main findings (called “primary reviews”). To examine and quantify the amount of overlap across the included reviews at the synthesis step, included primary studies within each systematic review were compared to evaluate the comprehensiveness of each review and duplication in the included primary literature. Finally, overlap was visually presented in tables and figures.

The findings of the Pollock study [19] suggest that eliminating systematic reviews may lead to loss of information. However, without specific empirical testing, it is not known whether the Patnode [27] overview suffered from a loss of information from eliminating low quality reviews. If Patnode [27] had included all systematic reviews then older, less comprehensive, and low-quality systematic reviews would have been included, thus introducing untrustworthy evidence into the results of the overview. By limiting their findings to high quality and comprehensive systematic reviews, Patnode and colleagues [27] gain efficiency in the overview production, reduce human resources needed to synthesise a large number of overviews, and produce an overview that is potentially more readable and useable.

#### 3) Murphy et al. self-management interventions in chronic obstructive pulmonary disease

We describe the overview by Murphy [23] that aimed to determine the clinical effectiveness of self-management interventions for adults with chronic obstructive pulmonary disease (COPD). Self-management interventions were defined as “structured and personalized, and often multi-component, with goals of motivating, engaging and supporting the patients to positively adapt their health behaviors and develop skills to better manage their disease” [44]. Murphy et al. used five methods, across the four steps in the conduct of an overview, to manage the overlap in 165 unique primary studies across the 16 included systematic reviews (Table 2). To manage overlap of primary studies, called “crossover” by the authors, they first included all studies based on pre-determined eligibility criteria. During data extraction and assessment of risk of bias stage, overlap across systematic reviews was assessed (Table 2). In the case of substantial overlap (over 70%), the higher quality review (using R-AMSTAR [45]) was selected if it was published the same year or more recently than the comparison reviews.

Overlap of primary studies was visually presented in tables. Overlap was calculated as the proportion of primary studies from one systematic review found in another, however this was not explicitly stated in the methods. In not explicitly reporting how overlap, or crossover, was calculated, reproducibility is jeopardized. Furthermore, the authors do not report the reference review for calculation of percentage overlap. Without knowledge of the reference review, percentage overlap is not reproducible [46]. Finally, the table reporting the overlap of RCTs across reviews has no legend to guide the reader in its interpretation. Given the multiple methods for managing overlap, authors of overview should explicitly and entirely state methods used in calculation and assessment of overlap.

Murphy et al. excluded two systematic reviews for high overlap (Bentsen and Harrison). The higher quality (and thus included) reviews (Zwerink and Jordan) included significantly more primary studies.

In both cases, systematic reviews with a low number of primary studies (Bentsen et Harrison) were excluded in favour of systematic reviews with significantly more primary studies (Zwerink et Jordan). During data synthesis, the authors noted that meta-analysis at the overview level would be inappropriate given the high percentage of overall overlap found.

In summary, the management of overlap focused on the data extraction, assessment of risk of bias and synthesis stages whilst maintaining broad eligibility criteria. Transparent reporting of methods dealing with overlap are necessary to interpret and reproduce results of overlap in overviews [46]. In selecting the highest quality review with under 70% overlap, Murphy et al. minimize the impact of duplicate primary studies in their overview. By limiting the inclusion to current, high quality reviews, Murphy and colleagues risk loss of information (Pollock [19]), but gain in utility, efficiency, and requiring potentially less resources.

#### 4) Prousali et al. efficacy and safety of interventions to control myopia progression in children

This case study involves an overview evaluating interventions aimed at slowing the progression of myopia in children [24]. Prevention of blindness and visual impairment from myopia was prioritised by the World Health Organisation (WHO)‘s VISION 2020 campaign [47]. This overview identified 18 systematic reviews that synthesized the efficacy of a variety of interventions for myopia from 44 unique primary studies (1989–2016). Prousali et al. used five methods across the four steps in the conduct of an overview to address overlap (Table 2). Eligible reviews had to meet pre-defined eligibility criteria for inclusion, which included systematic reviews of primary studies enrolling children or adolescents ≤18 years of age with myopia. Reviews without systematic search strategies or risk of bias assessment of primary studies were excluded. Interventions had to be optical or pharmacological and compared to single vision glasses, contact lenses or placebo. Primary outcomes were myopia progression and axial elongation.

A citation matrix was presented that identified primary studies that were included in more than one review. Overlap was quantified at the review level (as opposed to the outcome level) using the CCA [13] and high overlap was defined as equal to or more than 10%. If a review contained high overlap, reviews were retained that were (1) the most recent, (2) contained the highest amount of information (not defined by the authors), and (3) had the lowest risk of bias using ROBIS [48] and GRADE [49]. A meta-analysis was conducted using the unique primary studies included in the reviews. Overlap was considered moderate in this overview and was estimated at 6.2% using the CCA method. Since overlap was estimated at below 10%, all included reviews were retained in the analysis.

The authors used the CCA to assess overlap and state that it was “moderate” without explaining how moderate overlap may affect the overview’s findings. The authors then go on to perform a new meta-analysis, thus ignoring their overlap analysis, and removing the challenge of overlap from the equation. While conducting a ‘de novo’ meta-analysis eliminates the problem of very high or very low overlap, it is more resource intensive, may not always be feasible, and may introduce indirectness (i.e. lack of applicability) as the primary studies were not screened against the overview’s primary objective and PICO eligibility criteria [50]. When there are significant differences between the PICO of the overview and the PICO of the primary studies included in the systematic reviews, certainty in the evidence decreases. A better strategy would have been to conduct a systematic review with the aim to include primary studies directed related to the Prousali's research question, thus eliminating any problem of indirectness.

#### 5) Ryan et al. interventions to improve safe and effective medicines use by consumers

In this updated Cochrane overview of interventions to improve safe and effective medicines use by consumers [25], the authors searched the Cochrane database of systematic reviews (CDSR) and the Database of Abstracts of Reviews of Effects (DARE [51];). DARE contains summaries of methods and conclusions of included systematic reviews including a quality assessment. The authors deal with overlap at the eligibility criteria step using two methods, and one method at the assessment of risk of bias step (Table 2). Although it is common for Cochrane overviews to include only Cochrane reviews, Ryan et al. did not impose this restriction. All Cochrane reviews meeting the overviews inclusions criteria were included, and non-Cochrane reviews were excluded if they had substantial duplication of content with Cochrane reviews and were of low quality. Non-Cochrane reviews were included that were not within the PICO scope covered by Cochrane reviews. The rationale for prioritising inclusion of Cochrane reviews over non-Cochrane reviews was that Cochrane reviews are regularly updated and are of higher quality [52–55].

Quality of the included systematic reviews was rated using two scales; the Centre for Reviews and Dissemination assessment as part of the DARE, and the author’s assessment as judged using AMSTAR [43]. If reviews were classified as low quality with DARE or AMSTAR (≤4 out of 11 possible points), they were excluded. Further, non-Cochrane reviews with more than a half of their studies already included in a Cochrane review were excluded. At the synthesis stage, Ryan et al. retained one Cochrane review with unique content thus eliminating any issues related to overlapping primary study data (i.e. analysis of one high quality and comprehensive systematic review using decision rules (Table 2)).

There are two major advantages to including only relevant Cochrane reviews in an overview. First, quality, recency and comprehensiveness is higher in Cochrane reviews when compared to non-Cochrane reviews [52–55]. Second, according to the Cochrane policy, two reviews cannot be published on the same topic, resulting in a decreased risk of overlapping primary studies across reviews. Third, an empirical study found that selecting a Cochrane systematic review, as opposed to the most recent or highest quality non-Cochrane systematic review, maximized the amount of outcome data included in an overview [19], making that overview more relevant and useful for decision makers. However, including only Cochrane reviews of RCTs may limit data from highly relevant qualitative or quantitative reviews of observational studies.

#### 6) Thabet et al. clinical and pharmacokinetic/dynamic outcomes of prolonged infusions of beta-lactam antimicrobials

Thabet et al. conducted an overview investigating the comparative efficacy of prolonged infusions beta-lactam antibiotics compared to traditional intermittent infusions in hospitalized patients with infection. The authors used four methods across three steps in the conduct of an overview to address overlap (Table 2). At the eligibility step, eligible systematic reviews must have systematically searched the literature and synthesised clinical outcomes that included mortality or clinical cure. At the data extraction step, all included systematic reviews were data extracted. Quantification and assessment of overlap occurred at the synthesis step using citation matrices and by calculating the CCA at the outcome level across all included reviews and between each pairs of reviews.

**Fig. 3:**
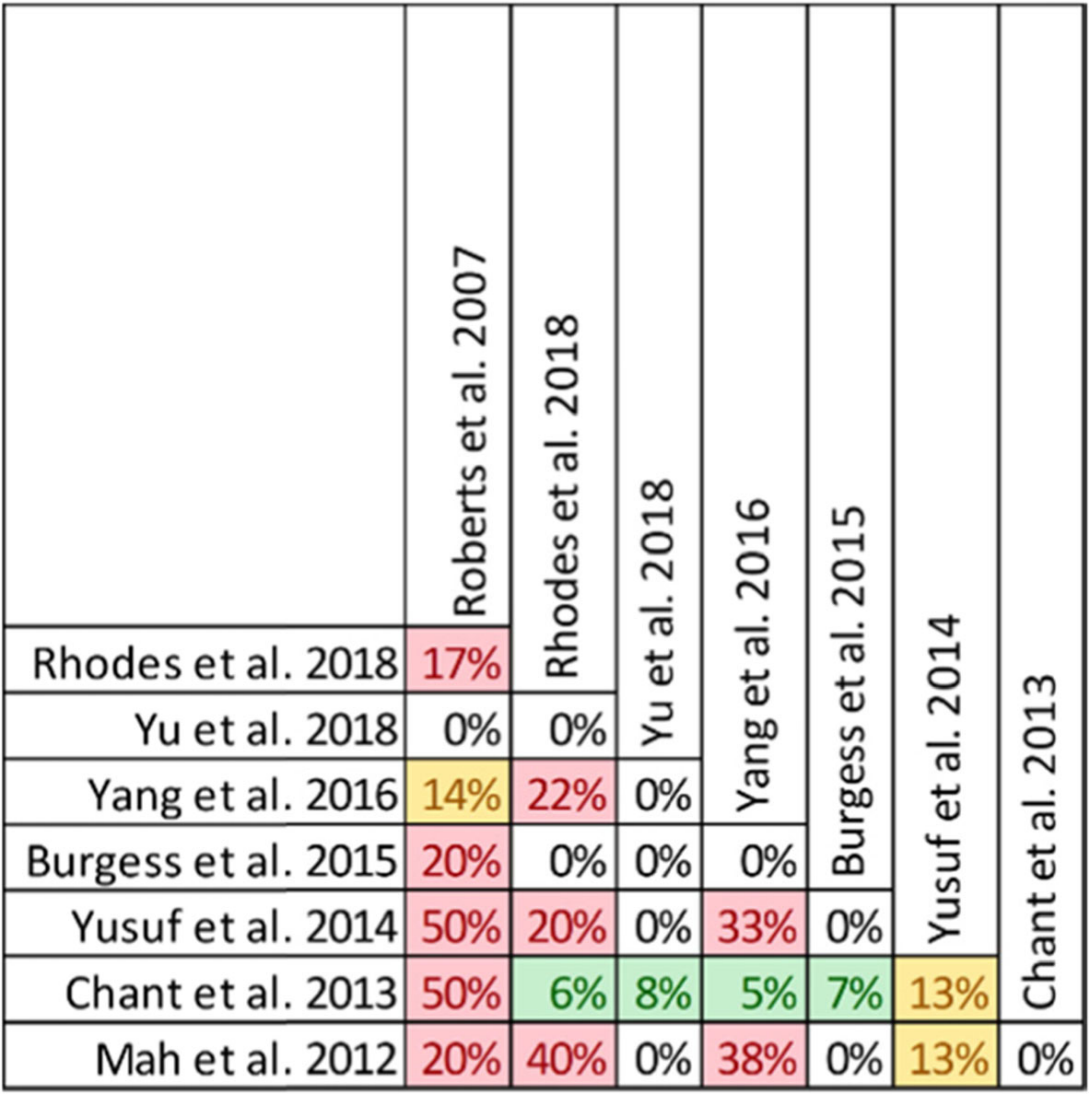
CCA calculations for pairs of reviews. Overlap categorization: 0–5% - slight (white), 6–10% - moderate (green), 11–15% - high (yellow), > 15% - very high (red)

**Fig. 4:**
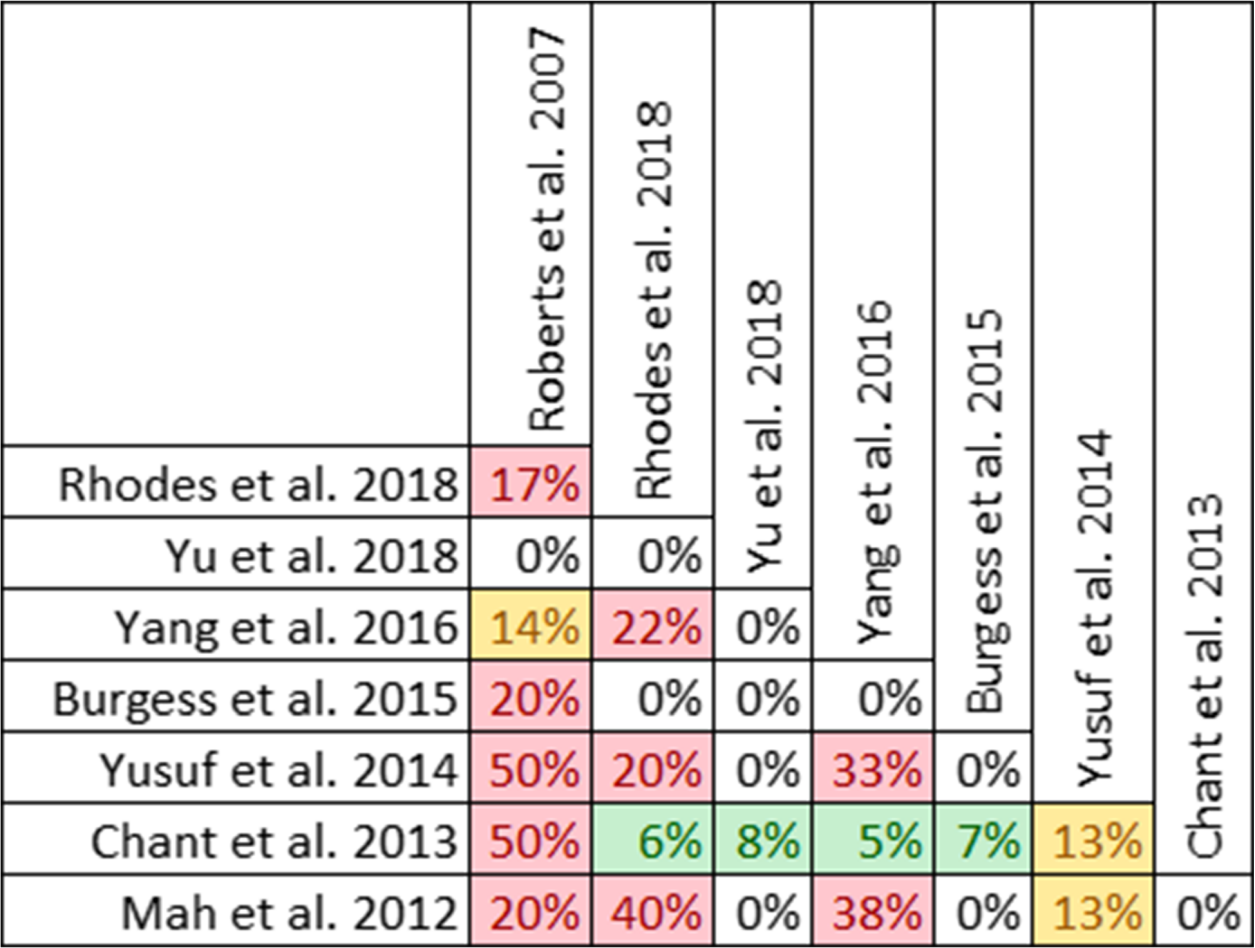
Citation matrix. Green indicates a trial included in a review, red indicates a trial excluded or omitted from a review and black indicates that the dates of publication make a trial ineligible for inclusion in a review

Twenty-one reviews involving 71 primary studies were included. For each of 9 clinical outcomes, a matrix of primary studies was created. Unique and overlapping studies were colour coded and overlap that was impossible due to publishing dates (i.e., primary study published after the systematic review) were also identified. Overlap was quantified using the CCA calculation across reviews for each outcome. Overlap thresholds, as determined by Pieper et al. [13], were used for interpretation of measured overlap (0–5% - slight, 6–10% - moderate, 11–15% - high, > 15% - very high). To further characterize overlap by outcome, CCA calculations for pairs of reviews were performed and presented as a grid (Fig. 3). Thabet et al. found that overlap was moderate to high for each outcome and percentage of unique references ranged from 38 to 78%. The authors suggest that the pairwise CCA grid (Fig. 3) allows the reader to identify which combinations of paired reviews had the highest overlap, while the citation matrix (Fig. 4) allows the reader to see which primary studies were common among reviews. The citation matrix also helped the authors understand why some studies were not identified by multiple reviews.

In this overview, the authors explain how both high overlap and low overlap influenced the findings of the overview. By including all the systematic reviews identified by the search, data mapping exercises identified significant variability between reviews with respect to scope, quality and findings. For example, when two reviews had similar findings and low overlap for a particular outcome, the results of the reviews could be trusted despite differences in PICO scope (i.e. different populations or beta-lactams) or differences in dates of publication (i.e. publication dates spanned several years between reviews). Alternatively, when low overlap was found between reviews with inconsistent and discordant findings, discrepancies were most often attributed to differences in scope or differences in dates of review publication.

In this example, the authors chose to use the CCA and citation matrices to assess the influence of overlap on their overview findings. While this approach allows for a more comprehensive data mapping exercise it also results in the potential for more reviews to be included where discrepancies in data from primary studies and discordant findings/conclusions of authors need to be evaluated and explained.

A potential limitation of this approach is the complicated analysis of overlap by outcome. With nine clinical outcomes considered and overlap assessed for each, it may make for a cumbersome and long read for the clinician who is more interested in the bottom line. However, a complicated analysis of overlap will lead to more comprehensive and reliable findings. When deciding whether to undertake an overview, authors should take into consideration the known or anticipated trade-offs of using different overlap methods. More empirical research about the trade-offs associated with alternative overlap methodological approaches is needed.

## Discussion

Our study underlines that there is no standard methodological approach to deal with overlap in primary studies across reviews. The level of complexity when dealing with overlap can vary depending on the yield, trends and patterns of the included literature and the scope of the overview question. Choosing a method might be dependent on the number of included reviews and their primary studies. For example, visual presentations of overlap becomes more challenging with an increasing number of reviews. In reviews with high yields, the breadth and depth of analysis can be challenging and resource intensive. Creating large reports with too much information and data can limit the readability and utility of an overview for decision makers and healthcare providers, and decrease the efficiency in its production.

Several possible approaches to manage overlap have been illustrated by presenting six case studies. The methodological approaches can be categorized by the stages in the conduct of an overview. For example, at the eligibility step, the trade-off of authors choosing one systematic review among many is a loss of potentially important information, which may lead to greater uncertainty about the effects of the intervention, while at the same time removing the issue of overlap. Including all systematic reviews is likely to introduce overlap, and will lead to challenges when synthesising a large amount of review data (e.g. resolving discrepant quality assessments, standardising effect metrics). When including all systematic reviews, resolving these challenges is likely to be resource intensive and cumbersome for the reader. More importantly, ignoring overlap in primary study data from the included reviews may affect the trustworthiness of the overview findings. If overlap is not addressed at the inclusion or data extraction steps, overview authors are advised to quantify and assess the influence of overlap at the synthesis stage of the overview.

As a general rule, we think the creating citation matrices are helpful. Many authors find that a citation matrix is essential to interpret the amount of overlap (e.g. using the CCA). However, better reporting of the reference review when calculating overlap, and details about how overlap is calculated is needed. Visual examination of citation matrices of primary studies included in reviews can be used to determine if low overlap is related to temporal gaps in search time frames, gaps in research topics, or how studies are clustered. Smaller citation matrices by outcome can be developed, which will aid in determining if overlap is an issue at the outcome level. More sophisticated methods to present overlap to the readers can be found in Bougioukas and colleagues [33], such as upset plots, heatmaps and node-link graphs for visualizing overlap.

Interpretation of the CCA has been an issue for many authors. First, CCA calculations for all primary studies across reviews, CCA calculations between 2 reviews only, and CCA calculations for one outcome [17] yield vastly different results. It may be the case as in Thabet et al. that pairs of systematic reviews had high overlap but the overview at a whole resulted in low or moderate overlap. Conducting all three overlap analyses can provide insight into which pairs of systematic reviews and outcomes have low overlap, thus helping authors highlight areas of trustworthy evidence but is more resource intensive.

When high overlap is found at the outcome level, only a few methods can be used to explain or resolve overlap. Examining potential reasons for different results or conclusions across reviews with high overlap can be highly informative and may resolve the issue. When overlap is still present and presents a problem, one review many be chosen for the overview synthesis. The limitations of this choice is that the one review may not represent the totality of evidence on the topic, and a loss of data may result. Statistical methods (e.g. conduct sensitivity analyses, inflate the variance of the pooled meta-analysis estimate) can also be used to manage overlap in primary studies across reviews, although these methods have not been frequently used in practice.

In general, there is a lack of empirical evidence testing these methods. We identified one study empirically evaluating the impact of five inclusion decisions on the conduct of an overview. The authors found that when overviews contain overlapping primary studies, selecting a Cochrane systematic review, as opposed to the most recent or highest quality non-Cochrane systematic review, maximized the amount of outcome data included in the overview [19]. While this study makes an important contribution to the empirical methods literature for overviews, significant gaps exist in evaluation of the methods used in the majority of steps and sub-steps in the conduct of an overview, especially methods used to resolve overlap. Evaluations of methods can provide evidence, which allows researchers to make informed choices about the most appropriate methods to use when conducting a study.

### Strengths and limitations of this study

Due to resource limitations, one author screened the studies titles/abstracts, full-text articles against the eligibility criteria, and extracted methods data relating to overlap. We recognise this is limitation of our study, as relevant citations could have been missed, and data errors could have been introduced. We only searched one database for relevant studies, but because (a) two of the authors are experts in overview methods, and follow the literature closely, and (b) we did forward citation searching on a seminal paper from 2014 on overlap in overviews [13], we feel like we have captured all relevant papers on this topic.

## Conclusions

Nine studies were found and mapped to the methods identified by the MOoR framework to manage overlap. Six studies were articles describing methods for overviews, two were guidance documents, and one was an empirical study. Several methods map across four steps in the conduct of an overview – the eligibility criteria step, the data extraction step, the assessment of risk of bias step, and the synthesis step. The methods can be used across the steps in the conduct of an overview depending on the nature and scope of the topic. No one standardised methodological approach exists to visualise, quantify and resolve overlap in primary studies across reviews.

Gaps in in evaluation of methods to address overlap were found and further investigation in this area is needed. Evaluation of the methods used in overviews is important as policymakers and clinicians need to be confident that the methods used to conduct overviews result in valid and reliable evidence.

## Supplementary Information

**Additional file 1.**

## Data Availability

All data generated or analysed during this study are included in this published article.
