# Supplementary material for: Managing overlap of primary study results across systematic reviews: practical considerations for authors of overviews of reviews
Source: BMC Med Res Methodol. 2021 Jul 7;21:140. doi: 10.1186/s12874-021-01269-y (PMC8265144; doi:10.1186/s12874-021-01269-y)
Supplement: Supplementary file 1 — Additional file 1. [file 12874_2021_1269_MOESM1_ESM.docx]

**Appendix A**

**PubMed Search strategy**

Search dates were from January 2016 to March 2020

(method*[TI] OR meta-epidemiol*) AND

((overview$ or review or synthesis or summary or cochrane or analysis) and (reviews or meta-analyses or articles or umbrella)).ti. or "umbrella review".ab. or (meta-review or metareview).ti,ab. or ((overview$ or reviews) and (systematic or cochrane)).ti. or (reviews adj2 meta).ab. or (reviews adj2 (published or quality or included or summar$)).ab. or "cochrane reviews".ab. or (evidence and (reviews or meta-analyses)).ti.

**Appendix B**

**Table A.1: Methods identified in the MoOR framework to deal with reviews that include *overlapping* information and data (e.g. arising from inclusion of the same primary studies)**

| **Step in the conduct of an overview** | **Methods for dealing with challenges** |
| --- | --- |
| Specification of purpose, objectives and scope | Not applicable |
| Eligibility criteria  1.4.2  1.5 (1.5.1–1.5.4) | 1.5 Determine eligibility criteria to deal with SRs with overlap  ▪ Include all SRs that meet the PICOs, irrespective of overlap  ▪ Select one SR from multiple addressing the same question using pre-specified quality or methodological criteria  1.4.2 Select SRs that meet minimum quality criteria or take a particular methodological approach. Minimum quality criteria include:  (i) meets definition of an SR, (e.g. explicit search)  (ii) up-to-date  (iii) quality of the SR (e.g. based on selected criteria; cutoffs derived from AMSTAR score)  (iv) use of best practice methods (e.g. specific RoB tools; Cochrane or AHRQ’s EPC methods)  (v) free of conflicts of interest (e.g. no industry funding)  (vi) reports sufficient primary study characteristics to interpret results (e.g. PICO elements, RoB assessment)  Methodological approaches include:  (vii) type of included primary studies  (viii) type of data  (ix) type of synthesis (e.g. meta-analysis, narrative)  ▪ Select one SR from multiple addressing the same question using pre-specified decision rules (e.g. combine one or more eligibility criteria in an algorithm)  ▪ Exclude SRs that do not contain any unique primary studies, when there are multiple SRs |
| Search methods | Not applicable |
| Data extraction  1.2,  2.2 (2.2.1, 2.2.2) | 1.2 Determine the data required to assess which SRs address the overview question and allow assessment of the overlap across SRs  2.2 Determine how overlapping information across SRs will be handled  2.2.1 Extract information from all SRs  2.2.2 Extract information from only one SR based on a priori eligibility criteria |
| Assessment of the risk of bias of SRs and primary studies  2.1.1 | 2.1.1 Report RoB assessment of primary studies from the included SRs, using the approaches specified for data extraction to deal with missing, flawed assessments, or discrepant assessments of the same primary study (i.e. where two or more SRs assess the same study using different tools or report discrepant judgements using the same tool; e.g. report RoB assessments from the highest quality SR (Jadad 1997 [6]) |
| Synthesis and presentation and summary of findings  1.1.2  5.0 | 1.1.2 Determine criteria for selecting SR/MA results, where SR/MAs include overlapping studies  1.1.2 Use decision rules or tools (e.g. Jadad tool) to select results from a subset of SR/MAs  5.0 Plan how to deal with overlap of primary studies included in more than one SR  ▪ Determine methods for quantifying overlap  ▪ Determine how to visually examine and present overlap of the primary studies across SRs  ▪ Determine methods for dealing with overlap  ▪ Use decision rules, or a tool, to select one (or a subset of) MAs with overlapping studies  ▪ Use statistical approaches to deal with overlap  ▪ Acknowledge overlap as a limitation |
| Assessment of the certainty of evidence  1.1.1  1.1.2  1.1.3  1.1.4  1.1.5 | 1.1.1 Assess the certainty of the evidence using a method developed for use in overviews  1.1.2 Assess the certainty of the evidence using an ad hoc method developed for a specific  overview  1.1.3 Report assessments of certainty of the evidence from the included SRs, using the approaches  specified for data extraction to deal with missing data, flawed or discordant assessments  (e.g. where two SRs use different methods to assess certainty of the evidence or report  discordant assessments using the same method)  1.1.4 Assess the certainty of the evidence using a method adapted or developed for use in overviews (e.g. assess certainty of the evidence taking into account the overlap in primary studies included in multiple SRs)  1.1.5 Assess the certainty of the evidence using an existing method customised to the overview |
